# Posterior vascular anatomy of the encephalon: a comprehensive review

**DOI:** 10.1007/s00276-024-03358-1

**Published:** 2024-04-23

**Authors:** Diego Morales-Roccuzzo, Mohammadmahdi Sabahi, Michal Obrzut, Edinson Najera, David Monterroso-Cohen, Shadi Bsat, Badih Adada, Hamid Borghei-Razavi

**Affiliations:** Department of Neurological Surgery, Pauline Braathen Neurological Center, Cleveland Clinic Florida, 2950 Cleveland Clinic Blvd., Weston, FL 33331 USA

**Keywords:** Neurovascular anatomy, Vertebral artery, Basilar artery, Posterior circulation, Vascular compendium, Brainstem

## Abstract

**Purpose:**

This article presents a comprehensive exploration of neurovascular anatomy of the encephalon, focusing specifically on the intricate network within the posterior circulation and the posterior fossa anatomy; enhancing understanding of its dynamics, essential for practitioners in neurosurgery and neurology areas.

**Method:**

A profound literature review was conducted by searching the PubMed and Google Scholar databases using main keywords related to neurovascular anatomy. The selected literature was meticulously scrutinized. Throughout the screening of pertinent papers, further articles or book chapters were obtained through additional assessment of the reference lists. Furthermore, four formalin-fixed, color latex–injected cadaveric specimens preserved in 70% ethanol solution were dissected under surgical microscope (Leica Microsystems Inc, 1700 Leider Ln, Buffalo Grove, IL 60089, USA), using microneurosurgical as well as standard instruments, and a high-speed surgical drill (Stryker Instruments 1941 Stryker Way Portage, MI 49002, USA). Ulterior anatomical dissection was performed.

**Results:**

Detailed examination of the basilar artery (BA), a common trunk formed by the union of the left and right vertebral arteries, denoted a tortuous course across the basilar sulcus. Emphasis is then placed on the Posterior Inferior Cerebellar Artery (PICA), Anterior Inferior Cerebellar Artery (AICA) and Superior Cerebellar Artery (SCA). Each artery’s complex course through the posterior fossa, its divisions, and potential stroke-related syndromes are explored in detail. The Posterior Cerebral Artery (PCA) is subsequently unveiled. The posterior fossa venous system is explained, categorizing its channels. A retrograde exploration traces the venous drainage back to the internal jugular vein, unraveling its pathways.

**Conclusion:**

This work serves as a succinct yet comprehensive guide, offering fundamental insights into neurovascular anatomy within the encephalon’s posterior circulation. Intended for both novice physicians and seasoned neuroanatomists, the article aims to facilitate a more efficient clinical decision-making in neurosurgical and neurological practices.

## Introduction

This work conceptualizes encephalic circulation as a dual inlet-single outlet vascular system: characterized by two parallel arterial tandems, namely the pair of Internal Carotid Arteries (ICAs) and the pair of Vertebral Arteries (VAs) that together form the Vertebrobasilar System. The ICAs contribute to the anterior circulation, while the VAs govern the posterior circulation. The primary venous outlet is facilitated by the pair of Internal Jugular Veins (IJVs) [18, 21, 36, 41].

This intricate arrangement is carefully designed so that each set of arteries is responsible for irrigating specific regions: the wide and structurally rich Supratentorial Space is supplied by the ICA, whereas the more densely arrayed Posterior Fossa is under the jurisdiction of the VA. In the latter, a “Rule of 3” for the posterior fossa intricately correlates three portions of the brainstem, three fissures, three cerebellar peduncles, three cerebellar cortical surfaces, and three cranial nerve groups. These are all supported by three respective arteries and accompanying veins, forming three complexes (upper, middle, and lower c.), with distinct segments and collateral branches intricately woven into the posterior circulation’s intricate tapestry which will be thoroughly elucidated later on [3, 38–40, 52].

**Fig. 1:**
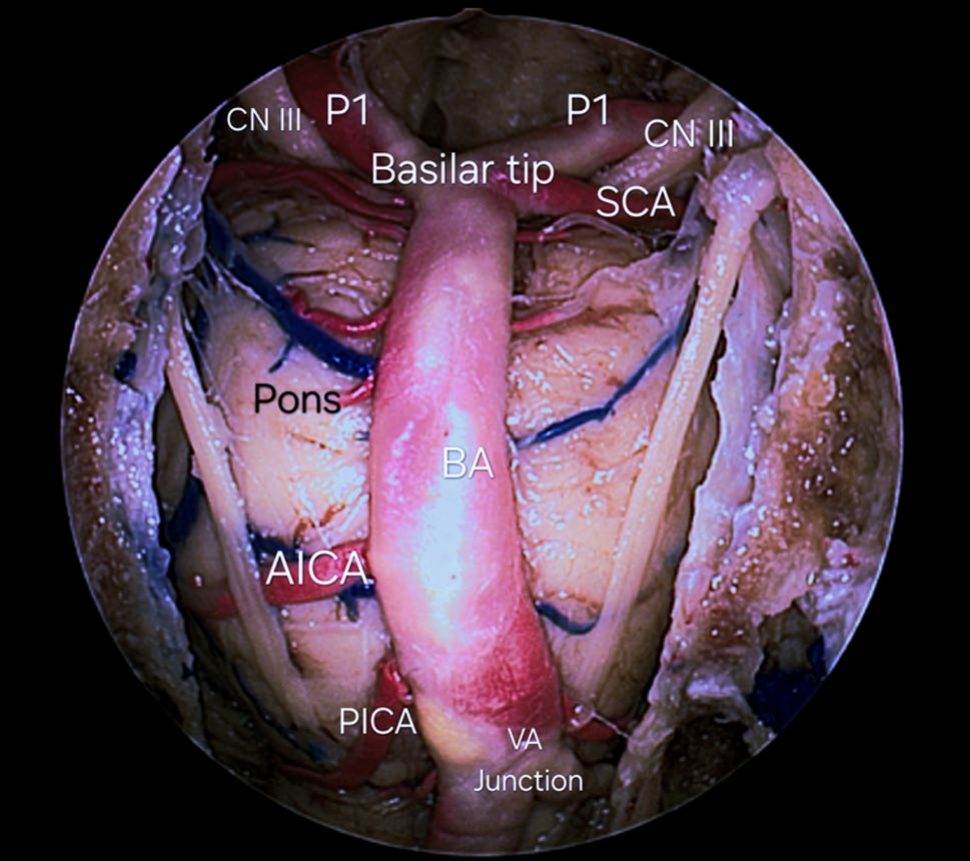
Basilar Artery (BA) anatomy from endoscopic endonasal transclival view: The left and right Vertebral Arteries (VA) junction can be observed, along with collaterals and perforator arteries going into the pons. *AICA* Anterior inferior cerebellar artery, *PICA* Posterior inferior cerebellar artery, *P1* Posterior cerebral artery, first segment, *SCA* Superior cerebellar artery

Various lesions, such as tumors, cavernous malformations, aneurysms, arteriovenous malformations, and dural arteriovenous fistulas, commonly impact the intracranial infratentorial compartment. This is particularly evident in the posterior cranial fossa or craniovertebral junction, where reduced spatial compliance can pose challenges [14, 52]. The effective management of these lesions, whether through surgical or endovascular interventions, heavily depends on a comprehensive understanding of the normal neurovascular anatomy of the posterior circulation [24, 25, 32]. Additionally, knowledge of the cisternal anatomy in the infratentorial region and its proximity to cranial nerves is crucial in surgical procedures, as these serve as corridors and landmarks for accessing specific segments of the vertebrobasilar system [20, 25, 48, 50]. This study aims to offer a concise overview of the microneurosurgical anatomy of the brain’s posterior circulation, an essential foundation for addressing tumors and neurovascular pathologies affecting the intracranial infratentorial region. [7, 13, 35] (Fig. 1).

Overall, this paper serves as a valuable tool in the rationale and preliminary study of neurovascular anatomy, both in clinical and research/academic environments. Aiming to be a firsthand aid to physicians and scholars in neurosurgical and neurological practice.

## Embryology

### Arterial embryology

Embryology of the encephalic vascular system must be the starting point of the current study to facilitate its understanding. Furthermore, we develop it in a straightforward and concise manner. During the fourth week of gestation, the central nervous system initiates the development of its vascular structures, coinciding with the establishment of the neural tube. Several longitudinal, low-caliber vessels supply the neural tube through simple diffusion. The development of the aortic arch and great vessels occurs in two phases including the brachial and post-brachial phases [18, 21].

As the first and second aortic arches regress, the third arch, known as the carotid arch, gives rise to the internal carotid artery (ICA). The fourth aortic arch becomes the aortic arch itself, persisting into adulthood and forming the right subclavian artery. The ICA divides into the cranial division, becoming the anterior cerebral artery (ACA), and the caudal division, becoming a precursor for the posterior communicating artery (PCommA). The anterior choroidal artery arises from the cranial division of the ICA, supplying blood to the telencephalon, while the caudal division gives rise to the superior cerebellar artery, providing blood to the cerebellum. In the hindbrain, the vertebrobasilar system is formed through the conjugation of the longitudinal neurovascular system, supplying the developing brain, spinal cord, vertebral bodies, and dura mater, and giving rise to the future vertebral artery (VA). Additionally, carotid-vertebrobasilar anastomoses provide temporary arterial supply from the ICA to the longitudinal neural artery, the future vertebrobasilar artery in the hindbrain, with four known types: trigeminal, otic, hypoglossal, and proatlantal intersegmental arteries. In some cases, persistence of the carotid to vertebrobasilar anastomosis is discovered in adulthood, considered a vestige of the corresponding primitive embryonic vessel [21].

### Venous embryology

The veins can be categorized into the supratentorial, basal, and infratentorial venous systems [18, 29, 30]. The supratentorial venous system is further divided into superficial and deep venous systems. Superficial and deep Sylvian veins comprise embryological superficial and deep telencephalic veins that initially drain into the primitive tentorial sinus. The deep venous system initially forms as a drainage pathway of the choroid plexus system, nourishing the early neural tube, with drainage through the median vein of the prosencephalon at 6–11 weeks of gestation. After the regression of this vein, the adult deep venous system is established. Parenchymal venous drainage either flows to the superficial veins through the superficial medullary veins or to the deep venous system (subependymal vein) through the deep medullary veins [18].

The basal system develops embryologically after the occlusion of the primitive tentorial sinus posteriorly, giving rise to the basal vein of Rosenthal. This vein receives both deep venous drainage and superficial venous drainage, serving as the superficial vein of the skull base.

The infratentorial system mainly consists of three routes draining the brainstem and cerebellum: anterior drainage (superior petrosal drainage) drains the archicerebellum through the embryological ventral metencephalic vein (trigeminal vein). Superior drainage (Galenic drainage) drains the paleocerebellum. Posterior drainage (torcular drainage) drains the neocerebellum. Similar to the cerebrum, the cerebellum has superficial and deep venous systems [30].

## Vertebral artery

**Fig. 2:**
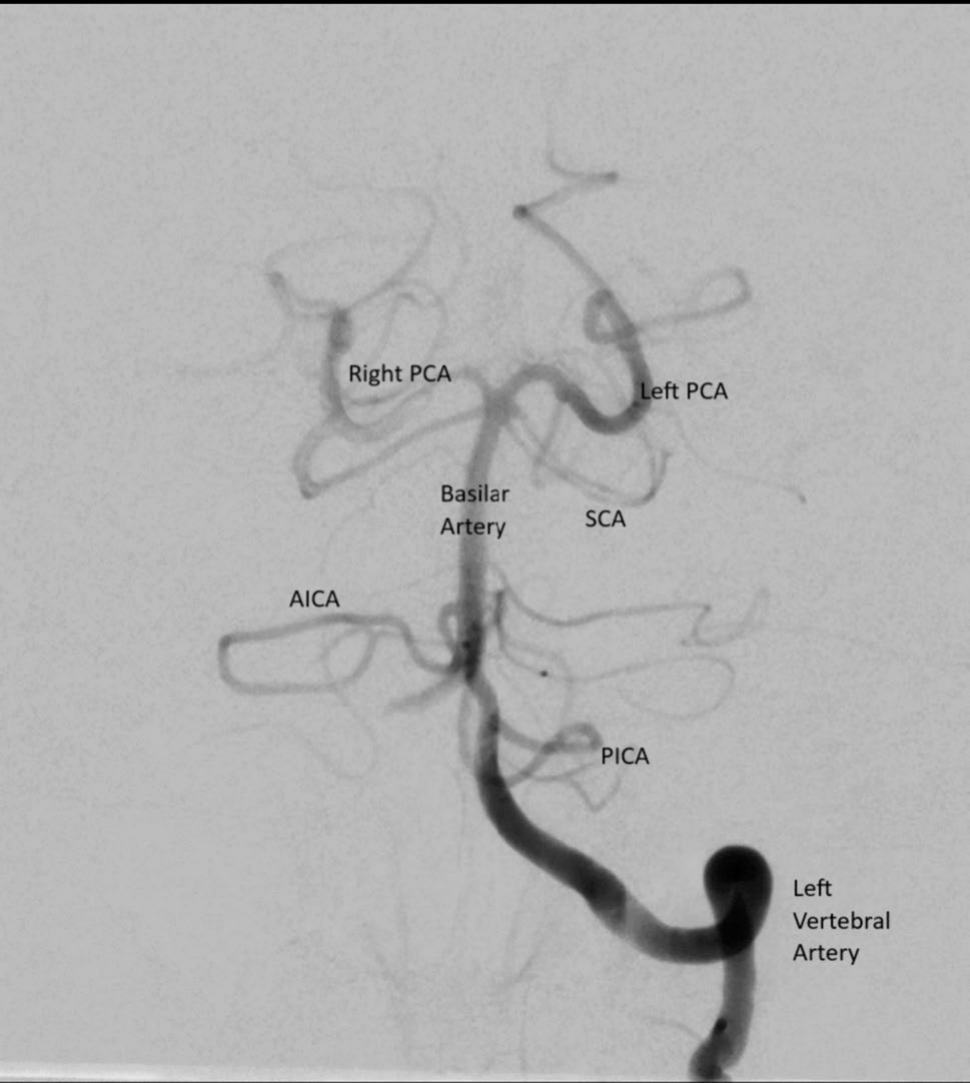
Posterior circuit angiogram (AP view). *AICA* Anterior inferior cerebellar artery,  *PCA* Posterior cerebral artery, *PICA* Posterior inferior cerebellar artery, *SCA* Superior cerebellar artery

**Fig. 3:**
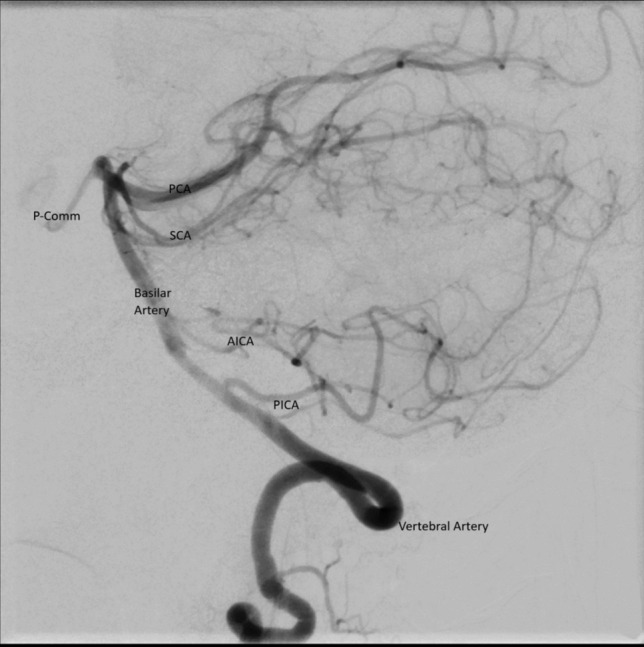
Posterior circuit angiogram (Lateral view). *AICA* Anterior inferior cerebellar artery, *PCA* Posterior cerebral artery, *PICA* Posterior inferior cerebellar artery, *P-Comm* Posterior communicating artery, *SCA* Superior cerebellar artery﻿

The VA is a paired artery, and it is the origin of the posterior circulation, also known as vertebrobasilar system. Each one of the two VA merge into a single, larger trunk, known as Basilar trunk, or Basilar Artery (BA), thus providing blood flow to the posterior fossa with all its anatomical complexity. The VA is the first and usually the largest branch of the subclavian artery. It has an average of 3 mm of diameter and the left VA is dominant in more than 60%. The right VA is hypoplastic in 10%. When a lesion occludes its flow, a vascular dissection occurs, or even in ischemic stroke, there can be found several signs and symptoms encompassed into syndromes such as Dejerine, Vernet and Jackson’s Syndromes, which usually comprise an ipsilateral lower cranial nerve deficit (weakness of soft palate, larynx, and pharynx) and a contralateral motor weakness or palsy. In some cases, different pathologies can produce even more serious consequences, potentially leading to vertebrobasilar insufficiency. This may either result in mild-moderate symptoms such as vertigo, ataxia, diplopia, or  posterior circulation strokes in severe cases, affecting large portions of brainstem and cerebellum, and potentially involving offspring arterial territories such as the Basilar Artery’s and either one or more of its branches [16]. This has catastrophic socioeconomic consequences and an appalling impact on morbidity and mortality. In this realm of pathologies, to aid in diagnosis and treatment, endovascular neurosurgery plays a fundamental and indispensable role (Figs. 2, 3).

Four segments of the VA are individualized [5, 21, 38].

### V1—prevertebral

This segment (pre-foraminal) commences when the VA emerges from subclavian artery, within the triangle of the VA, delimited by the longus colli and anterior scalene muscles, and the first part of the subclavian artery. Afterwards courses superiorly and posteriorly and enters the foramen transversarium, usually of the sixth vertebral body. As clinical hint, thrombosis in the V1 segment might result in reduced blood flow to the upper cervical spine and the posterior inferior cerebellum.

### V2—intraforaminal

It ascends in cephalad direction through the transverse foramina (hence its name, foraminal) of the cervical vertebrae surrounded by sympathetic fibers derived from the stellate ganglion, along a venous plexus that intimately surrounds it. It is located anterior to the cervical nerves’ roots. Finally, it then bends laterally to enter the foramen within the transverse process of the axis, in a superolateral trajectory.

### V3—suboccipital

V3 leaves the foramen of the axis and curves posteriorly and medially in a groove on the upper aspect of the Atlas’ posterior arch, lying behind the C1 lateral mass, and then enters the foramen magnum medial to the atlanto-occipital joint. This portion, also called atlantic part, lies anatomically in the deep posterior plane of the neck, and can be found in Tillaux’s Triangle (suboccipital triangle) [1], a space limited by three muscles: the oblique capitis inferior (inferior side), the oblique capitis superior (superior-lateral side) and the rectus capitis posterior major (superior-medial side). It also should be noted that C1 nerve root runs beneath the artery and over on the posterior arch of the atlas. In the interior of the triangle, all neurovascular structures are enclosed by the posterior atlanto-occipital membrane, forming the floor, and a thick connective tissue layer, which closes Tillaux’s triangle posteriorly forming its roof. As minor clinical note, a thrombosis in the V3 segment can have severe consequences, potentially leading to vertebrobasilar insufficiency. In this regard, the daunting clinical significance when discussing the anatomy of this segment, it is crucial to note its division into three distinct portions. This division holds significance particularly in the context of utilizing this segment for revascularization procedures, as each portion requires specific surgical approaches.

The V3 segment can be delineated into three portions: (a) the vertical portion, located between the transverse processes of the first and second cervical vertebrae; (b) the horizontal portion, which courses within the groove on the upper surface of the posterior arch of the atlas; and (c) the oblique portion, which traverses through the dura mater. Emerging from the third segment of the vertebral artery are the posterior meningeal arteries, posterior spinal arteries, muscular branches, and occasionally the posterior inferior cerebellar arteries [31, 45].

### V4—intradural

The final division becomes intradural once it pierces the dura (in a location somewhat variable) and immediately enters the subarachnoid space; additionally, this portion is known as intracranial. Finally, it merges the contralateral homologous at the vertebral confluence located at the lower pontine border to form the BA. If one considers the pre-olivary sulcus, V4 can be further divided into lateral medullary and posterior medullary. The rootlets of the C1 nerve are surpassed by the lateral medullary segment, and from that point it ascends forward anteriorly to the dentate ligament and spinal accessory nerve (XI CN). Among the collateral branches, the Posterior Inferior Cerebellar Artery (PICA) is the most relevant intracranial-wise and has the most complex and tortuous course, but the following can be listed as constant branches of the VA: anterior and posterior meningeal arteries, anterior and posterior spinal arteries and PICA.

### Posterior inferior cerebellar artery (PICA)

The PICA stands out as the largest, most crucial, and intricate branch. Typically originating 10 mm distal to the point where the VA transitions to intradural status, it appears within 20 mm proximal to the vertebrobasilar junction. In accordance with the Rule of 3 mentioned earlier, the PICA is closely associated with the medulla oblongata, the cerebellar-medullary fissure, the inferior cerebellar peduncle, the suboccipital surface of the cerebellum, and the lower cranial nerves (IX, X, XI, and XII). Regarding venous relations, the main deep vein within the fissure is the cerebello-medullary vein, coupled with veins of the cerebellar peduncles.

**Fig. 4:**
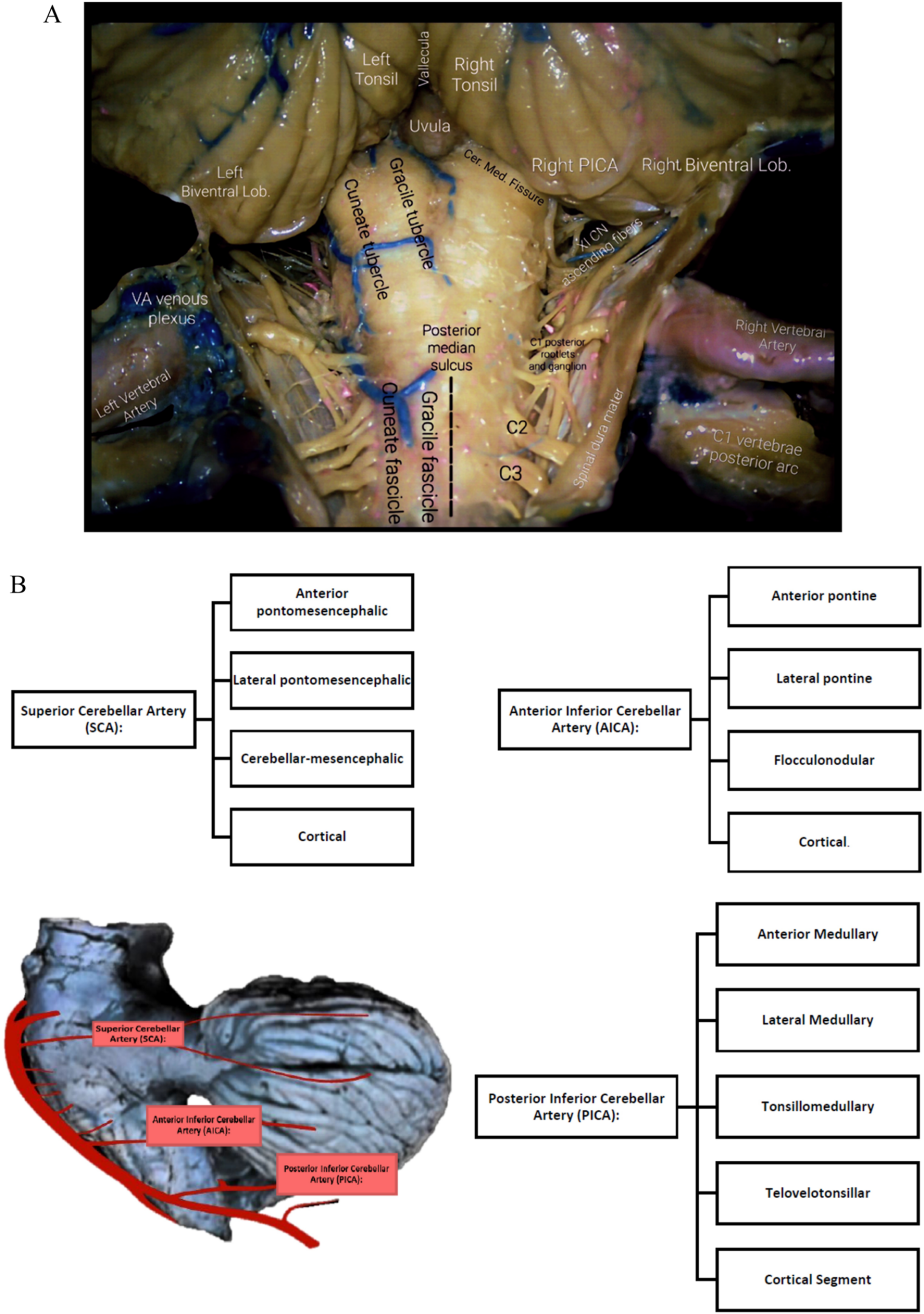
**A** In situ PICA cortical branches and posterior view of caudal cerebellum, dorsal brainstem and spinal cord. **B** PICA/AICA/SCA branches

Its emergence from the VA occurs in proximity to the inferior olive, and it follows a posterior trajectory around the medulla, typically navigating through the XII CN rootlets emerging from the pre-olivary sulcus, outlining a convoluted course [3, 13, 38, 51]. Moreover, it is important to note that in certain instances, it arises from an extracranial (hence, extradural) location within the V3 segment. Involvement of this artery in different pathology can produce a very flourished array of signs and symptoms, such as those found in the Wallenberg Syndrome (Ipsilateral ataxia, hemifacial thermo-anesthesia, weakness of soft palate, larynx, and pharynx, Horner’s ipsilesional; Contralateral Thermo-analgesia due to somatosensory tract involvement)[16]. It mainly correlates to the segment involved and the structures it supplies (Fig. 4A, B).

The PICA has five segments as follows:

#### Anterior medullary

This segment of the PICA extends from its origin at the VA to the inferior olivary eminence. It provides one or two short medullary circumflex branches destined to irrigation of the ventral medulla. This segment is not always present. Lesions in this segment which may impact the ventral medulla. Potential consequences could include motor and sensory deficits, as well as disruptions in autonomic functions associated with the medulla.

#### Lateral medullary

It extends from the most prominent point of the medullar olive to origin of IX, X and XI cranial nerves, giving up to five perforating branches that supply brainstem. On the contrary, this is a constant segment. As a commentary, involvement of this segment in pathology may cause the “Wallenberg syndrome” or lateral medullary syndrome.

#### Tonsillomedullary

The tonsillomedullary segment of PICA starts when the CN nerves are left behind and then extends medially to the medulla posterior aspect, close to the inferior half of the tonsil. Finally, it ends when the artery ascends through the medial surface of the tonsil, configuring the *caudal loop*, that coincides with the tonsil’s inferior pole.

#### Telovelotonsillar

The most complex segment commences when PICA ascends in through the medial surface of the tonsil and then reaches the inferior medullary velum. It ends when the vessel leaves the fissure between the tonsil and the cerebellar hemisphere, where it projects a new loop, the *cranial loop,* which coincides with the fastigium. This segment provides irrigation to the fourth ventricle choroidal plexus and the *tela choroidea.*

#### Cortical segment

It starts when PICA exits the fissure between the vermis and the tonsil and provides medial branches to the vermis and lateral branches to the suboccipital surface of the cerebellar hemisphere. It typically provides three constant branches including choroidal artery, tonsillohemispheric artery, and inferior vermian artery.

Although it falls beyond the scope of this work to clinically assess vascular topography, for the sake of elucidation, it can be pointed out that tonsillomedullary segment lesions may affect the tonsillomedullary region, potentially leading to disturbances in the coordination of swallowing, speech. Likewise, telovelotonsillar segment lesions might produce disturbances in the irrigation of the fourth ventricle choroidal plexus and the tela choroidea, thus resulting in changes in cerebrospinal fluid dynamics, potentially impacting intracranial pressure. Finally, cortical segment may affect the cerebellar hemisphere and vermis, leading to ataxia, dysmetria and intention tremor [16].

## Basilar artery

The BA is a common trunk formed by the union of the left and right vertebral arteries. It begins at the medullary-pontine sulcus and becomes narrowly related with the basilar apophysis of the occipital bone and the pontine cistern [48]. It has a somewhat tortuous course, as it slithers across the basilar sulcus, a midline vertical sulcus on the ventral aspect of the pons where the artery normally runs. Along its course it provides several pontine branches that irrigate numerous structures of the pons. Additionally, there are crucial collaterals to be found, which will be detailed shortly. At its end, the Basilar Artery terminates at a bifurcation right in front of the pontomesencephalic sulcus, point in which it gives off its two symmetrical terminal branches: the left and right posterior cerebral arteries, at the level of the interpeduncular cistern [25]. This division is also known as Basilar Apex or “Basilar tip”, which may be situated at the exact same level, above, or beneath the superior border of the dorsum sellae, towards the prepontine cistern. In cases where the basilar bifurcation lies above the dorsum sellae or below it, they are designated as high-riding or low-riding basilar tips, respectively, having different implications regarding the surgical approach of choice [7, 11, 25, 35]. Basilar lesions, such as injury due to arterial wall dissection can lead to a broad assortment of gravely severe outcomes, ranging from many brainstem alternate syndromes to the dreaded Locked-in Syndrome [10, 12, 16]. The BA has also an utmost importance when treating stroke or aneurysms via endovascular procedures [26, 32].

The branches of the BA are anterior inferior cerebellar artery (AICA), posterior cerebral artery (PCA), superior cerebellar artery (SCA), and pontine perforating branches which are responsible for supplying numerous and crucial pontine nuclei.

### Anterior inferior cerebellar artery (AICA)

The AICA is the first important collateral of the BA. It courses across the central portion of the cerebellopontine angle (CPA) and is part of one of the three complexes described by Rhoton [38, 39]: AICA is intimately associated with the pons, the lateral recess of the fourth ventricle, the middle cerebellar peduncle, the VI, VII and VIII cranial nerves, the cerebellopontine fissure, the cerebellar petrosal surface and the conspicuous cerebellar-pontine cistern [50]. Its major deep venous group lies in the cerebello-pontine fissure, accompanied by veins that receive drainage from the middle cerebellar peduncle. From the lower part of BA, it originates proximate to VI CNs origin and then runs posterolaterally anterior to VI, VII and VIII CN. It often makes a loop that runs across the internal auditory meatus. It commonly divides into a rostral and a caudal trunk, near the VII–VIII CN complex [13, 43, 44].

When involved in a stroke or lesion, should it happen, certain syndromes can occur: such as the Millard-Gubler or the Foville Syndrome, that include VII and VIII ipsilateral palsy and contralateral sensory-motor deficit. Even more, ataxic hemiparesis can be found, when the cerebellum blood flow is compromised. From this artery can also arise complex wide neck aneurysms prone to be treated surgically through intraoperative clipping [43], being high-risk procedures. The AICA is divided into four segments and each of these segments may include more than one trunk, depending on the level of bifurcation of the AICA.

#### Anterior pontine

In relation with the VI CN, the anterior pontine segment of AICA extends from its arising point at the BA to the medullary olive.

#### Lateral pontine

The lateral pontine segment of AICA extends to the anterolateral region of the pons, in which it continues its course towards the CPA. This segment can be further subdivided into three parts: premeatal, meatal and postmeatal, depending on the proximity and relation with the internal acoustic meatus. It gives rise to several nerve-related branches including the labyrinth or labyrinthine artery, the recurrent perforating arteries (in the parafloccular space or on the cisternal surface of the middle cerebellar peduncle [42], and the subarcuate artery. Moreover, specific branches for the cranial nerves from VI to the XI can be found, overlapping with blood flow sources as diverse as those coming from the PICA or even the ascending pharyngeal artery [9, 15].

#### Flocculonodular

The flocculonodular segment of AICA begins where the AICA passes the flocculus towards the CPA, until it finally reaches it, along with the cerebellopontine fissure and middle cerebellar peduncle. Perforating arteries can also be found nourishing the brainstem or cerebellum with the blood flow provided from this segment.

#### Cortical

It supplies the petrosal cerebellar surface and the floccular area. It bifurcates into superior and inferior branches. The superior branch, anastomoses with cortical branches of the SCA. The inferior branch, anastomoses with cortical branches of the PICA at a point that varies according to whether the AICA expresses cortical dominance over the latter or, on the contrary, the PICA dominates over the former.

### Superior cerebellar artery (SCA)

This artery originates 2 or 3 mm below the basilar bifurcation, travels immediately beneath the III CN, goes round the pontine-mesencephalic union, and passes below the IV CN and above the V CN in all cases. Afterwards, it reaches the cerebellar-mesencephalic fissure and finally heads towards the vermis and the tentorial cerebellar surface. SCA is part of the upper complex, and relates with the upper half of the fourth ventricle’s roof, the superior cerebellar peduncle, the cerebellar-mesencephalic fissure, the tentorial surface of the cerebellum, and the III, IV and V cranial nerves [13, 53]. As for venous anatomy of the upper complex, its key deep vein rests within the cerebello-mesencephalic fissure, as a functional tandem with veins that drain the superior cerebellar peduncle. The midbrain is frequently the portion of the brainstem to be related if this vessel is somehow subject to injury or occlusion. We point out as mere examples the Weber, the Claude or the Benedict syndromes (which involve ipsilateral III cranial nerve paralysis and contralateral weakness or ataxia). Although, SCA aneurysms are rare, their management alternatives are not well delineated and need to be tailored accordingly. As stated, there is an increasing role of endovascular treatment for all aneurysms, especially for aneurysms of the posterior circulation. Nevertheless, in some situations (wide base, dysmorphic features) coiling is not feasible, thus their surgical management has its own distinctive complexity and requires careful planning. When ruptured, these dysmorphic aneurysms must be treated urgently either via endovascular surgery or intraoperative clipping [24]. Four segments of SCA can be recognized as follows:

#### Anterior pontomesencephalic

The anterior pontomesencephalic segment of SCA extends from its origin in the BA to the anterolateral portion of the mesencephalon.

#### Lateral pontomesencephalic

The Lateral pontomesencephalic segment of SCA extends from the lateral portion of the mesencephalon until it enters the cerebellar-mesencephalic fissure, closely related to the fifth nerve.

#### Cerebellar-mesencephalic

The cerebellar-mesencephalic segment of SCA courses through the homologous fissure, along with IV CN.

#### Cortical

The cortical segment of SCA commences where the SCA leaves the fissure to reach its destination territories, the vermis and the tentorial cerebellar surface.

SCA’s collateral branches are perforating, pre-cerebellar and cortical [38]. Perforating collateral branches is accountable for irrigation the tegmentum, the interpeduncular fossa, the cerebral peduncle, the junction of the superior and middle cerebellar peduncles and part of the quadrigeminal plate. This perforating collateral include direct and circumflex types. The direct type pursues a straight course to enter the brainstem, while the circumflex type winds around the brainstem before terminating in it. Pre-cerebellar collaterals direct blood flow to the cerebellar central nodule and the superior medullary velum. Finally, the cortical collaterals are providing nourishment to the petrosal cerebellar surface and include Vermian branches, Hemispheric branches, and Marginal branch (Not constant). Seldom, a variant meningeal supply to the medial tentorium, arising from the superior cerebellar artery (SCA) which its believed anastomosed with the artery of Davidoff and Schechter (ADS—further discussed), later came to be known as the artery of Wollschlaeger and Wollschlaeger (AWW)[27].

### Posterior cerebral artery (PCA)

**Fig. 5:**
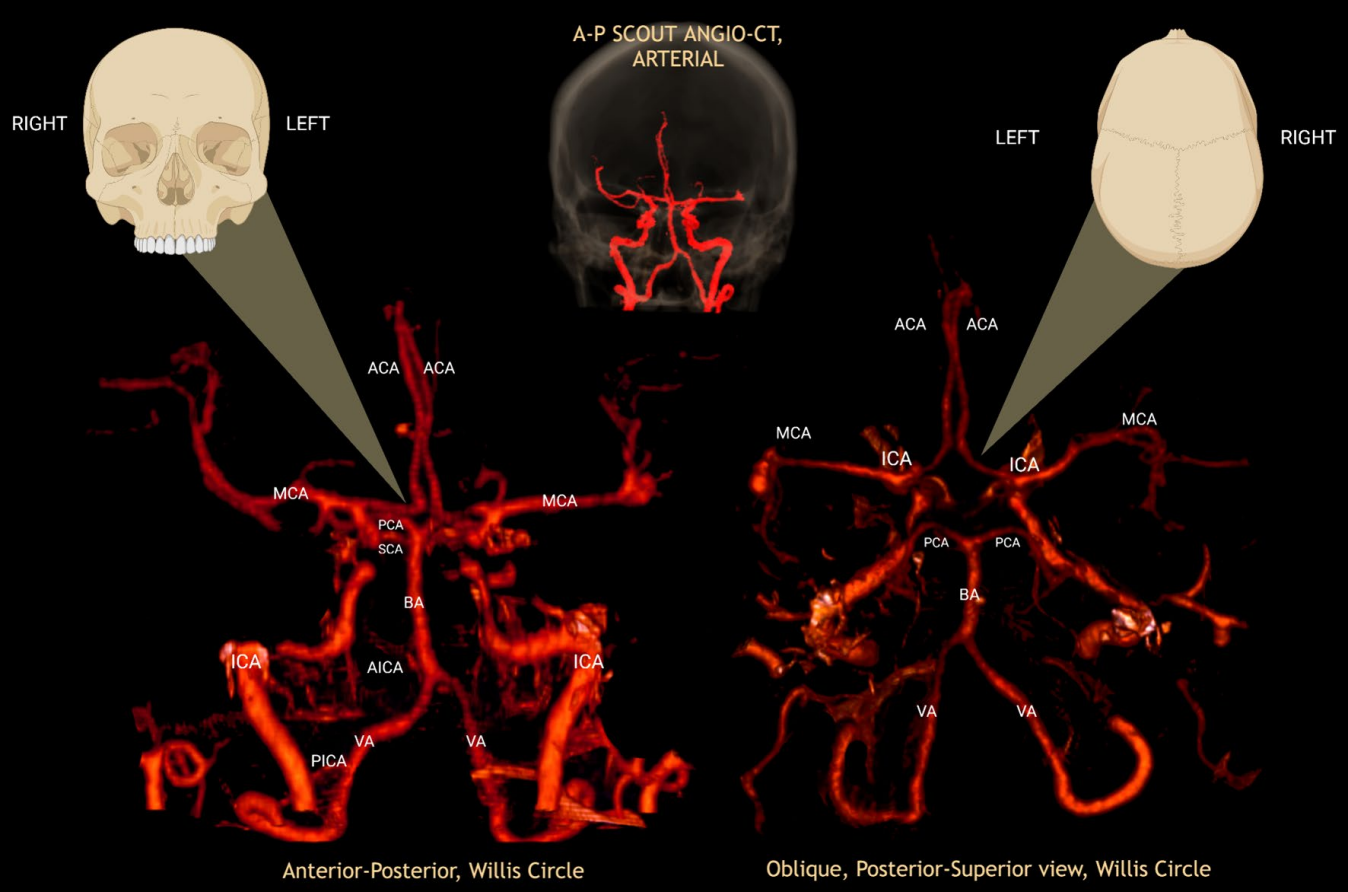
Angio-CT 3D render of arterial circulation of the brain, comprised by anterior and posterior circuits, with detailed view and labeling of the main vessels. In the oblique posterior-superior view, it is observed incidentally a dominant right Posterior Communicating Artery with a much greater caliber than its counterpart. *ACA* Anterior cerebral artery, *AICA* Anterior inferior cerebellar artery, *MCA* Middle cerebral artery, *PCA* Posterior cerebral artery, *PICA* Posterior inferior cerebellar artery, *SCA* Superior cerebellar artery, *VA* Vertebral Artery

**Fig. 6:**
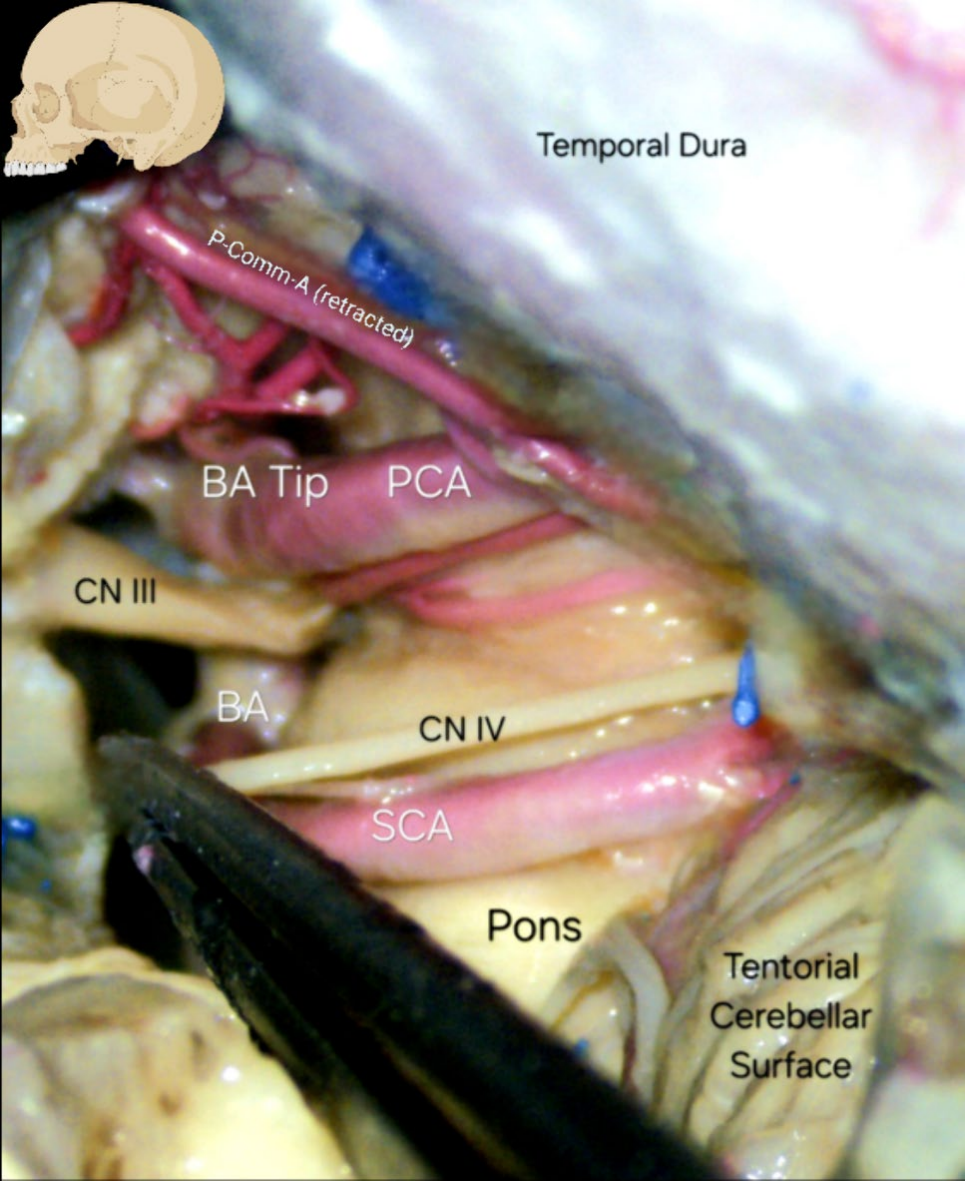
Anatomical dissection featuring the Basilar tip in the pre-pontine cistern at the vicinity of the interpeduncular and crural cisterns, and related neurovascular structures such as cranial nerves III and IV, as well as the Posterior Cerebral, Posterior Communicating and Superior Cerebellar Arteries

**Fig. 7:**
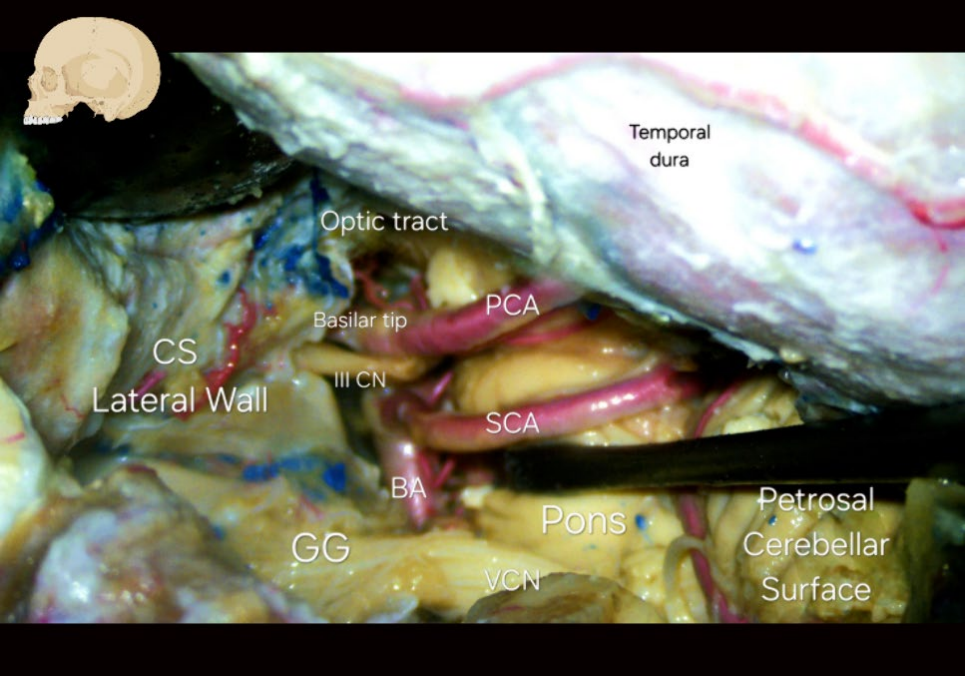
Basilar tip and Posterior Cerebral Artery's neural, cranial and cisternal relations; transcranial subtemporal view

The posterior half of the Circle of Willis reaches completion when the PCA, which arises from the basilar bifurcation below the posterior perforated substance, receives the Posterior communicating artery (PCommA), consequently closing the circuit. The PCA is joined by the PCommA at the lateral margin of the interpeduncular cistern, circles the brainstem passing through the crural and ambiens cisterns and reaches the quadrigeminal cistern, to finally distribute to the posterior part of the hemisphere. The basilar bifurcation may be found as far caudal as 1.3 mm below the pontomesencephalic connection and as far rostral as the mamillary bodies, sometimes even adjacent to the floor of the third ventricle. The BA usually bifurcates opposite the interpeduncular fossa, but some variations have been described [13, 17, 34, 49, 51, 53]. As a brief clinical note, depending on the segment or branch involved, a brimming and diverse set of sign and symptoms can be identified; for instance, Parinaud Syndrome (Paralysis of upgaze, convergence-retraction nystagmus, lid retraction, and light near dissociation) in cases of perforating branches and cortical blindness, when calcarine cortex (with visual eloquence) is the location of the pathology. Additionally, it can also be the origin of aneurysms of the Basilar Apex area, before joining the Posterior Communicating Artery (PCommA) or at its junction with the aforementioned vessel [32] (Figs. 5, 6, 7).

The PCA can be divided into four parts, P1 through P4:

#### P1 segment (pre-communicating)

Also referred to as pre-communicating segment, it extends from the basilar tip to the point in which the PCA is joined by the PCommA. An anatomic variant in 1/3 of hemispheres is known as fetal configuration, in which P1 has a smaller diameter than the PCommA (or even more, completely absent) and the PCA emerges from the ICA, hence supplying blood flow as well to the posterior circulation [26].

#### P2 segment (post-communicating)

This portion begins when the PCA is joint by the PCommA, lies within the crural and ambient cisterns, and terminates lateral to the midbrain. This segment is subdivided in two parts, according to its relationship with the cisterns: P2-A or crural segment, because it travels around the cerebral peduncle in the crural cistern, and P2-P or ambiens segment, because it courses lateral to the midbrain in the ambient cistern. The P2 segment runs through the crural cistern, relating itself to the cerebral peduncle, uncus, the optic tract and basal vein ambient cisterns, and then courses along the ambiens cistern, between the lateral midbrain and the parahippocampal and dentate gyri, in the vicinity of the optic tract, basal vein, geniculate bodies, the trochlear nerve and tentorial edge [13, 48–50].

#### P3 segment (quadrigeminal)

P3 courses dorsally from the posterolateral aspect of the midbrain and ambiens cistern to reach the quadrigeminal cistern and then terminates at the anterior limit of the calcarine fissure. The PCA here often divides into its major terminal branches, the calcarine and parieto-occipital arteries. The quadrigeminal segments from both sides approach each other posterior to the cerebral colliculi and the point where both PCAs are nearest is known as the quadrigeminal or collicular point [8].

#### P4 segment (cortical-calcarine)

It is majorly comprised of the branches distributed to the parieto-occipital and calcarine cortical surface. The anterior end of the calcarine sulcus marks the beginning of P4.

The PCA gives off three kind of branches, including central perforating branches, ventricular branches and cerebral branches, providing not only the posterior portion of the cerebral hemispheres, as its denomination suggests, but also sends critical branches to diencephalon, mesencephalon and ventricular system [2, 7, 8, 38, 51]. Central perforating branches divided into direct and circumflex perforating arteries, PCA gives off branches to the diencephalon and midbrain (Tectum, cerebral peduncles, and three nuclei including Edinger-Westphal, oculomotor and trochlear). Among these perforating arteries, one can commonly identify, Interpeduncular thalamoperforators, Mesencephalic perforating arteries, Thalamogeniculate arteries, Quadrigeminal and geniculate branches and Artery of Percheron which is a seldom anatomic variant that nourishes the paramedian thalami and the rostral midbrain bilaterally. It has also been identified a meningeal artery supplying the medial tentorium, arising from the posterior cerebral artery (PCA), the artery of Davidoff and Schechter (ADS), a vessel named in homage to its mentors in neuroradiology [27]. It appears to originate distal to the confluence of the PCA and posterior communicating artery (PCommA), suggesting that it arises from the P2 segment.

Ventricular branches destined for the choroid plexus and walls of the lateral and third ventricles as well as adjacent structures. Very often one or both of these branches join the *Anterior choroidal artery* in the proximity of the choroid plexus of the lateral ventricles. There are two constant branches including medial posterior choroidal, that arising mostly from P1 or P2 and lateral posterior choroidal, that arising from P2 in most cases.

**Fig. 8:**
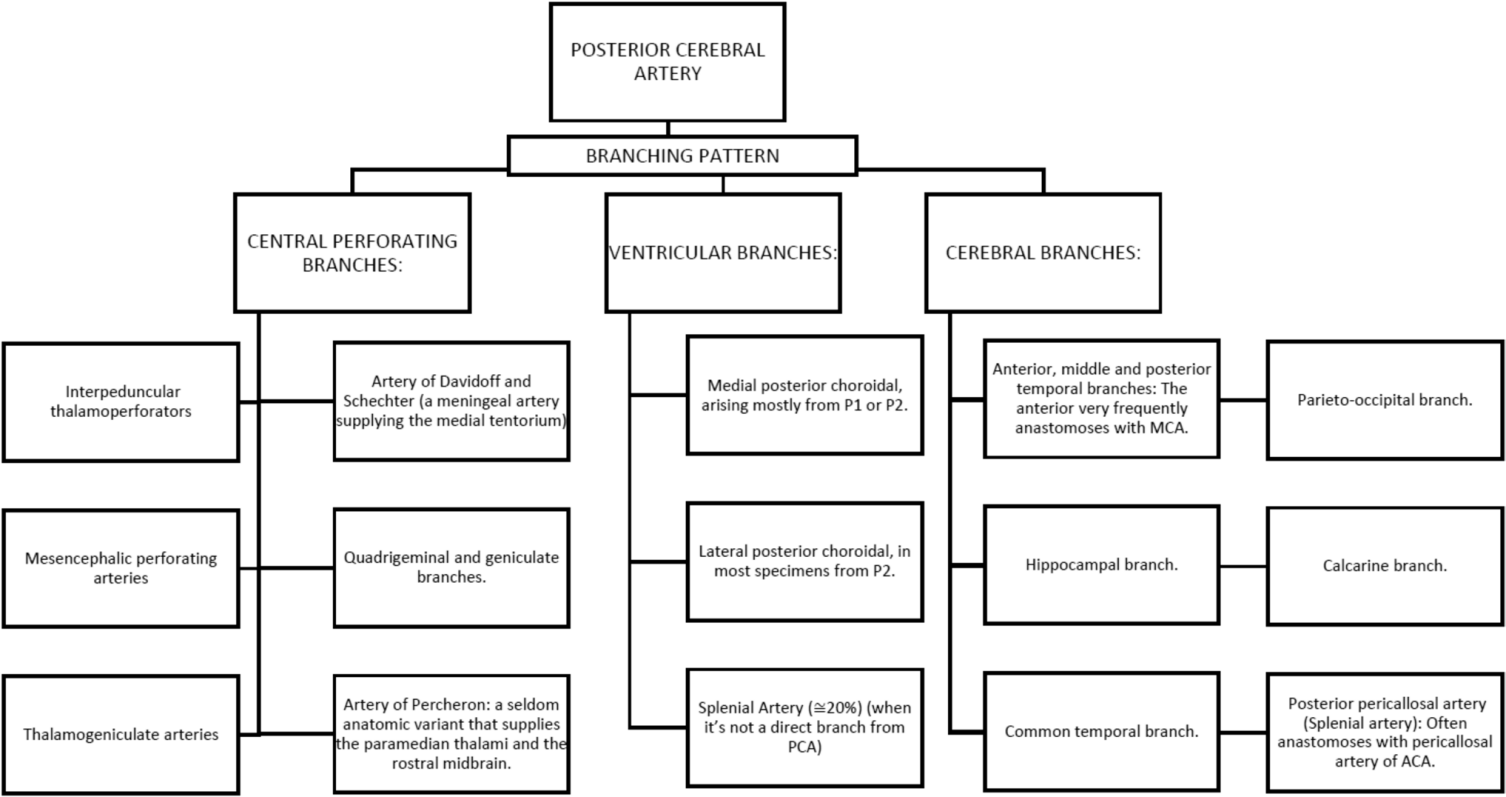
Posterior cerebral artery main branches

Cerebral branches are headed to and held accountable for the supply of the cerebral cortex and splenium of the corpus callosum. A wide cortical vascular territory is supplied by the numerous branches that emerge from the cortical segment. Amongst the constant collaterals, the following can be listed [28]: Anterior, middle and posterior temporal branches, in which the anterior branch frequently anastomoses with anterior temporal branch of MCA, Hippocampal branch, Common temporal branch, Parieto-occipital branch, Calcarine branch, and Posterior pericallosal artery (Splenial artery) which often anastomoses with pericallosal artery of ACA (Fig. 8).

## Posterior fossa venous system

Although the posterior fossa has been thoroughly described as a compartmentalized region divided into three neurovascular complexes, should one delve deeper into venous anatomy, focusing on functional anatomy and embryological origins, with absolutely no deleterious effect on the taxonomy previously presented, the veins of the posterior fossa could be categorized into four groups: superficial, deep, brainstem, and bridging veins. Superficial veins are further divided based on the cortical surfaces they drain: the tentorial surface by the superior hemispheric and superior vermian veins, the suboccipital surface by the inferior hemispheric and inferior vermian veins, and the petrosal surface by the anterior hemispheric veins. Major deep veins within fissures between the cerebellum and brainstem include those of the cerebellomesencephalic, cerebellomedullary, and cerebellopontine fissures, while veins on the cerebellar peduncles include those of the superior, middle, and inferior cerebellar peduncles. Brainstem veins are named according to whether they drain the midbrain, pons, or medulla and their course direction. Posterior fossa veins terminate as bridging veins, which collect into three groups: a galenic group draining into the vein of Galen, a petrosal group into the petrosal sinuses, and a tentorial group into the tentorial sinuses, ultimately emptying into the transverse, straight, or superior petrosal sinus [6, 37–39].

**Fig. 9:**
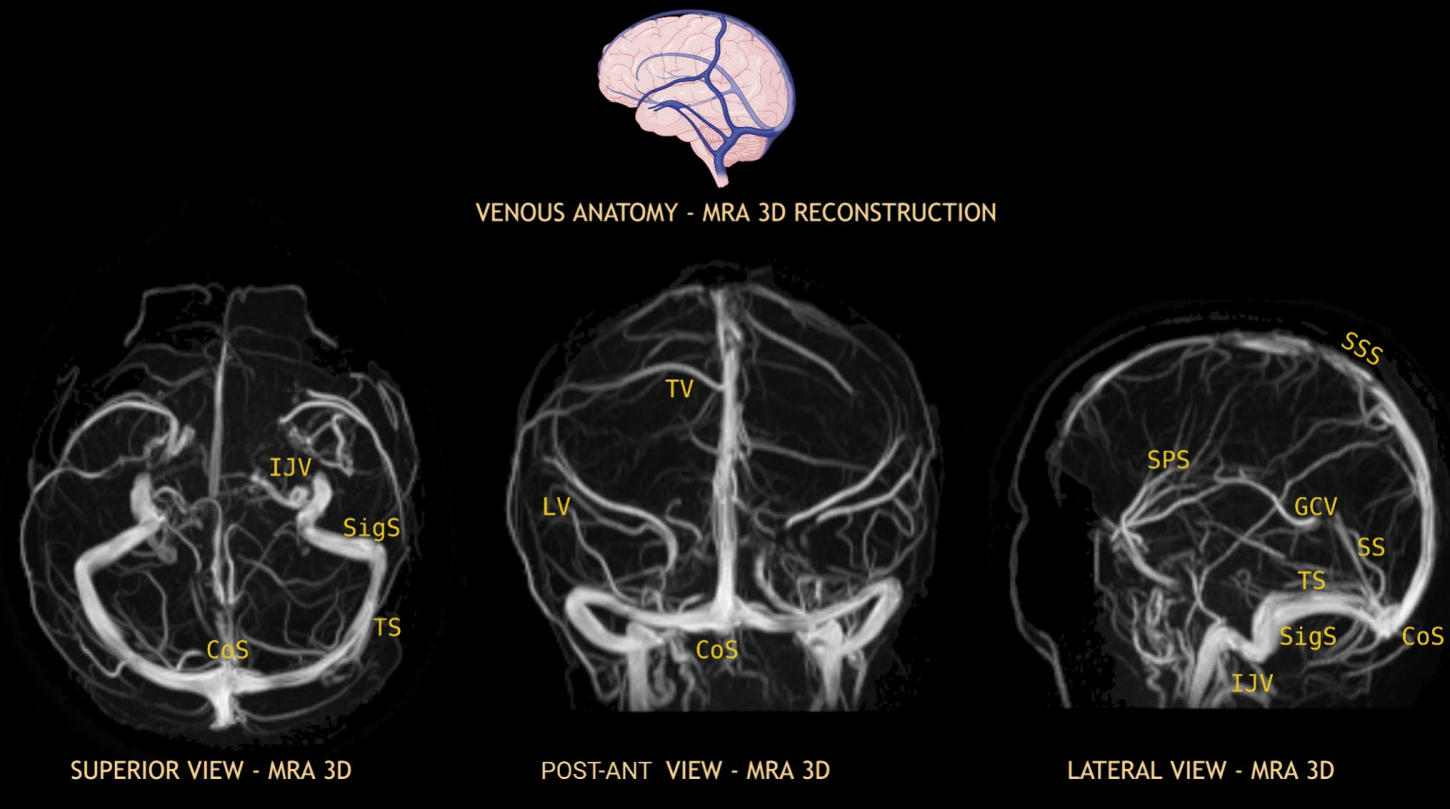
MR-angiography: 3D reconstruction of venous stage, that emphasizes venous stages and global anatomy of the dural venous sinuses. *CoS* Confluence of sinuses, *IJV* Internal jugular vein, *GCV* Great cerebral vein, *LV* Vein of Labbé, *SigS* Sigmoid sinus, *SPS* Sphenoparietal sinus, *SS* Straight sinus, *SSS* Superior sagittal sinus, *TS* Transverse sinus, *TV* Vein of Trolard

Ultimately, it cannot be overlooked that modern neuroimaging, such as Angio-CT and Angio-MRI, are essential for thoroughly studying the region. These methods provide real-time visualization of every neurovascular structure in situ, enhancing an ulterior understanding of the posterior fossa, in addition to the well-established Digital Subtraction Angiography (DSA) (Fig. 9).

In other words, the superficial veins drain the cortical cerebellar surface and are divided on the basis of whether they drain the tentorial, petrosal, or suboccipital surface and whether they drain the hemisphere or vermis [6, 13, 33]. On the other side of the spectrum, the deep veins course along the three aforementioned deep fissures between the cerebellum and brainstem close to the superior medullary velum and to the three cerebellar peduncles that lie within these fissures. Thus, they drain each mentioned region. The vein of the cerebellomesencephalic fissure arises from it and is intimately related to the superior half of the roof of the fourth ventricle; the vein of the cerebellomedullary fissure courses in this fissure and is intimately related to the inferior half of the roof; and the vein of the cerebellopontine fissure runs through it and is intimately related to the lateral recess and lateral walls of the fourth ventricle [33].

**Fig. 10:**
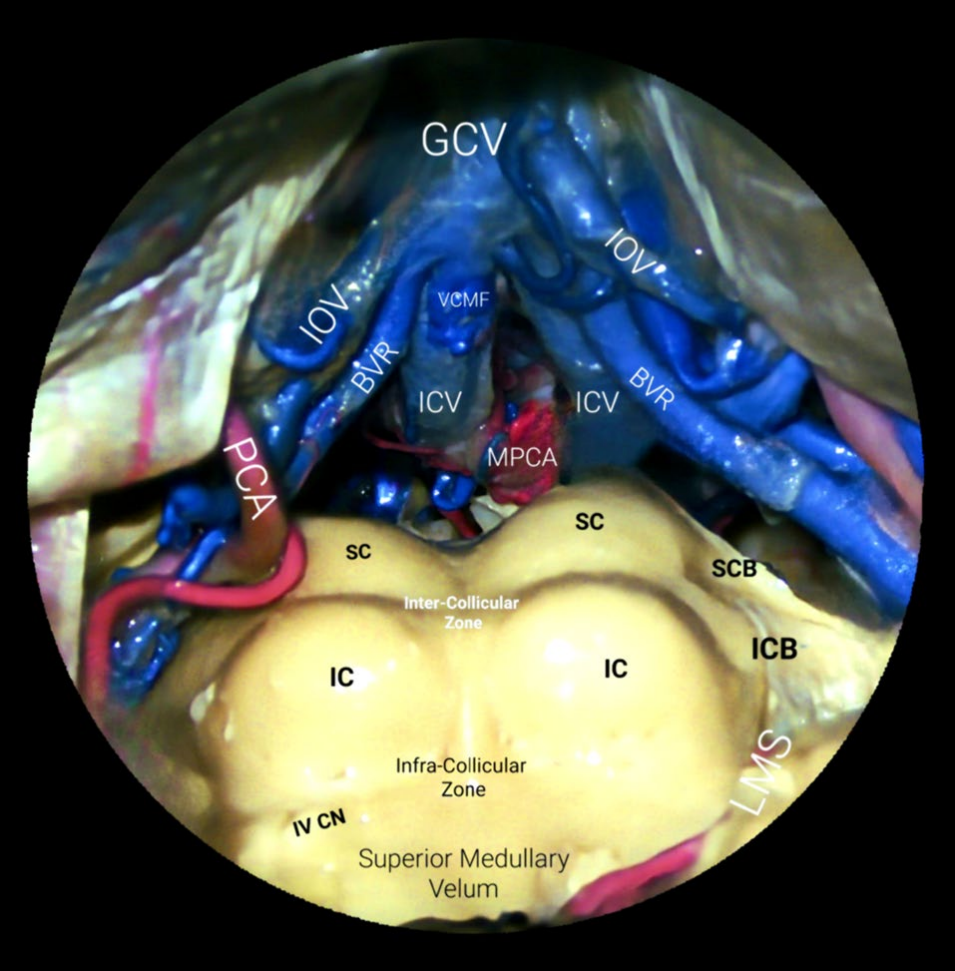
Great cerebral vein of Galen in situ and related anatomy in the quadrigeminal plate, posterior upper brainstem

As stated, regarding the veins of the brainstem, they are named based on three features: the portion of the brainstem drained (mesencephalon, pons, or medulla oblongata), the surface of the brainstem drained, and the direction in which they run (transverse or longitudinal) [33]. Finally, the terminations of the veins draining the brainstem and cerebellum form bridging veins that cross the subarachnoid and subdural spaces and reach the dural venous sinuses. These drainages collect into three groups, which include a superior or Galenic group that drains into the vein of Galen; an anterior or petrosal group that drains into the petrosal sinuses; and a posterior or tentorial group that drains into the sinuses converging on the torcula [23]. The latter group includes cerebellar hemispheric tentorial bridging veins, the neurosurgical clinical implications of which were thoroughly described by Prof. Uğur Türe and his team, among other colleagues [4, 22] (Fig. 10).

### Main veins and tributaries

The following section attempts to trail the global venous drainage of the brain: an interwoven network of cranial venous drainage that provides an escape route for deoxygenated blood high on carbon dioxide after it has carried oxygen and metabolic nourishment to brain parenchyma; and has additional functional implications such as maintaining intracranial pressure when confronted with trauma or space occupying pathologies occur. Beginning with the Internal Jugular Vein (IJV), it serves as a major conduit for venous outflow from the brain. The IJV receives blood from the Inferior Petrosal Sinus near its convergence with the Sigmoid Sinus. Moving further along the pathway, the Sigmoid Sinus continues the journey, accompanied by several important tributaries. Notably, the Superior Petrosal Sinus terminates at the Sigmoid Sinus within 1 cm of the of the junction of the Sigmoid and Transverse Sinuses, in the vicinity of the sino-dural angle. All of these pathways above mentioned receive a variable percentage of blood flow from the Basilar Venous Plexus and Petroclival Venous Confluence, marking a critical juncture in venous drainage [3, 6, 18, 19, 29, 37, 46, 47]. Towards a more cranial direction, the complex sellar and parasellar region takes ahold of significant structures such as the Cavernous Sinus, which is intricately connected to the surrounding anatomy. Correlated to this cavernous set of connections, one finds the Intercavernous Sinus, the Sphenoparietal Sinus, and the Ophthalmic Vein, all contributing to the intricate venous architecture.

The venous landscape is further characterized by variability, as seen in the Occipital Sinus, which exhibits a rather inconsistent presence across individuals. Worth addressing, as a clinical hint, is that the right Transverse Sinus assumes prominence, prevailing in more than 60% of cases. Notably, it receives blood from the Vein of Labbé, also known as the Inferior Anastomotic Vein.

The convergence of sinuses at the Torcula, or Confluence of Sinuses, receives the Lateral Sinuses (coupled Transverse and Sigmoid Sinus). From here, the Straight Sinus extends, leading to the Superior Sagittal Sinus, a major cerebral vein. This expansive structure accommodates cortical veins, the Middle Superficial Cerebral Vein, and the prominent Vein of Trolard, noted as the most prominent superficial vein on the non-dominant side, with Labbé assuming prominence on the dominant side [13, 37].

**Fig. 11:**
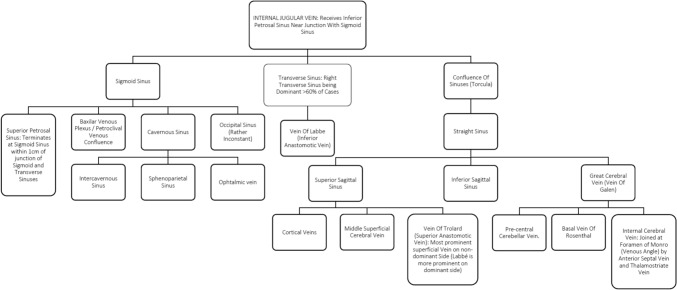
Venous outflow summary

Further downstream from the Straight Sinus, the venous pathway encompasses the Inferior Sagittal Sinus before culminating in the Great Cerebral Vein, also known as the Vein of Galen. This vein receives contributions from significant tributaries including the Pre-central Cerebellar Vein, Basal Vein of Rosenthal, and the Internal Cerebral Vein. Notably, the Internal Cerebral Vein joins at the Foramen of Monro, or Venous Angle, by the Anterior Septal Vein and the Thalamostriate Vein, further highlighting the intricacy of cerebral venous anatomy [6, 33, 37] (Fig. 11).

## Strengths and limitations

The current work on posterior circuit provides a succinct yet comprehensive overview of the fundamentals that every novice in neurovascular anatomy must know. For the sake of brevity, we have not included vertebral plexuses, External Carotid Artery or External Jugular Vein. Advanced vascular morphometry has not been discussed since the reader can expand through the reference list and cornerstone literature such as Prof. Rhoton’s collection of texts. The authors are well aware that several expertly crafted articles on this subject have been published. Nevertheless, we believe this paper adds up to those that preceded, and provides a straightforward approach to this complex subject, highlighting nuances of embryology, clinical implications and basic microneurosurgical anatomy. It is our belief that this work, while compelling, will not overwhelm the novel reader and trainees in neuroanatomy, neurology, neurointerventional radiology and neurosurgery residents and fellows.

## Conclusion

The meaning of this work is to serve as a preliminary but thorough guide, providing essential knowledge on the neurovascular anatomy of the encephalon, specifically on the posterior circulation. We believe it aids in the understanding of the intricate vasculature of the brain and posterior fossa, which is crucial for neurosurgical and neurology related practice regarding clinical decision-making. Hence, its main purpose is to provide an early framework, thus enhancing whatever initial knowledge novice physicians, neurosurgical and neurological trainees might have on that subject, henceforth making more efficient the use of available time. It is meant to be an abridgment to be kept in the armamentarium of training scholars and neuroanatomists.

## Data Availability

The authors confirm that the data supporting the findings of this study are available within the article.
